# Crystal structure of 3-methyl­pyridine-2-carbaldehyde 4-methyl­thio­semi­carba­zone monohydrate

**DOI:** 10.1107/S2056989015005034

**Published:** 2015-03-25

**Authors:** Nur Shuhada Mohd Mokhtaruddin, Thahira Begum S. A. Ravoof, Mohamed Ibrahim Mohamed Tahir, Edward R. T. Tiekink

**Affiliations:** a Department of Chemistry, Universiti Putra Malaysia, 43400 Serdang, Malaysia; b Department of Chemistry, University of Malaya, 50603 Kuala Lumpur, Malaysia

**Keywords:** crystal structure, hydrogen bonding, thio­semicarbazone

## Abstract

In the title hydrate, C_9_H_12_N_4_S·H_2_O (systematic name: 3-methyl-1-{(*E*)-[(3-methyl­pyridin-2-yl)methyl­idene]amino}­thio­urea monohydrate), a small twist is noted between the pyridine ring and the rest of the organic mol­ecule [dihedral angle = 6.96 (5)°]. The imine and pyridine N atoms are *syn*, and the amine H atoms are *anti*. The latter arrangement allows for the formation of an intra­molecular N—H⋯N(imine) hydrogen bond. Both the N-bonded H atoms form hydrogen bonds to symmetry-related water mol­ecules, and the latter forms O—H hydrogen bonds with the pyridine N and thione S atoms. These inter­actions lead to supra­molecular layers that stack along the *a*-axis direction with no specific inter­actions between them.

## Related literature   

For background to the coordination chemistry of thio­semicarbazones, see: Beraldo *et al.* (2001 ▸); Sreekanth *et al.* (2004 ▸). For the structure of the parent compound, in which the pyridine N atom is *anti* to the imine N atom, see: West *et al.* (1996 ▸). For the synthesis of the title compound, see: Ali *et al.* (1997 ▸).
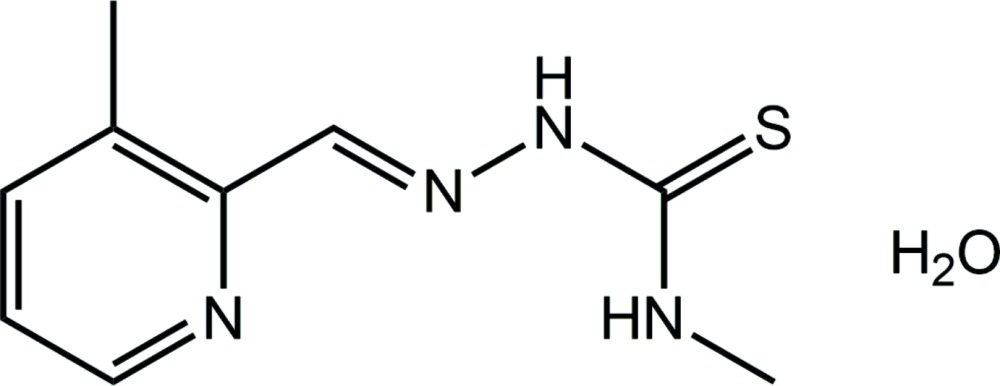


## Experimental   

### Crystal data  

C_9_H_12_N_4_S·H_2_O
*M* *_r_* = 226.30
Monoclinic, 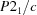

*a* = 10.4493 (3) Å
*b* = 13.6989 (3) Å
*c* = 8.0235 (3) Å
β = 102.816 (3)°
*V* = 1119.90 (6) Å^3^
*Z* = 4
Cu *K*α radiation
μ = 2.42 mm^−1^
*T* = 100 K
0.30 × 0.20 × 0.10 mm

### Data collection  

Oxford Diffraction Xcaliber Eos Gemini diffractometer
Absorption correction: multi-scan (*CrysAlis PRO*; Agilent, 2011 ▸) *T*_min_ = 0.860, *T*_max_ = 1.000
14591 measured reflections
2160 independent reflections
2044 reflections with *I* > 2σ(*I*)
*R*_int_ = 0.022

### Refinement  

*R*[*F* ^2^ > 2σ(*F* ^2^)] = 0.034
*wR*(*F* ^2^) = 0.097
*S* = 1.05
2160 reflections
150 parameters
5 restraints
H atoms treated by a mixture of independent and constrained refinement
Δρ_max_ = 0.31 e Å^−3^
Δρ_min_ = −0.25 e Å^−3^

### 

Data collection: *CrysAlis PRO* (Agilent, 2011 ▸); cell refinement: *CrysAlis PRO*; data reduction: *CrysAlis PRO*; program(s) used to solve structure: *SHELXS97* (Sheldrick, 2015 ▸); program(s) used to refine structure: *SHELXL2014* (Sheldrick, 2015 ▸); molecular graphics: *ORTEP-3 for Windows* (Farrugia, 2012 ▸) and *DIAMOND* (Brandenburg, 2006 ▸); software used to prepare material for publication: *publCIF* (Westrip, 2010 ▸).

## Supplementary Material

Crystal structure: contains datablock(s) 1, I. DOI: 10.1107/S2056989015005034/hb7381sup1.cif

Structure factors: contains datablock(s) I. DOI: 10.1107/S2056989015005034/hb7381Isup2.hkl

Supporting information file. DOI: 10.1107/S2056989015005034/hb7381Isup3.cml

**Fig. 1.**
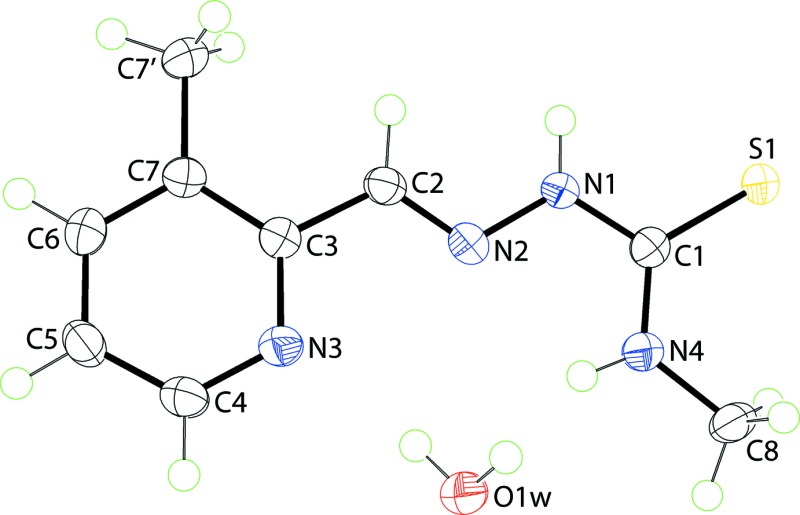
The molecular structure of the title compound showing the atom-labelling scheme and displacement ellipsoids at the 70% probability level.

. DOI: 10.1107/S2056989015005034/hb7381fig1.tif
The mol­ecular structure of the title compound showing the atom-labelling scheme and displacement ellipsoids at the 70% probability level.

**Fig. 2.**
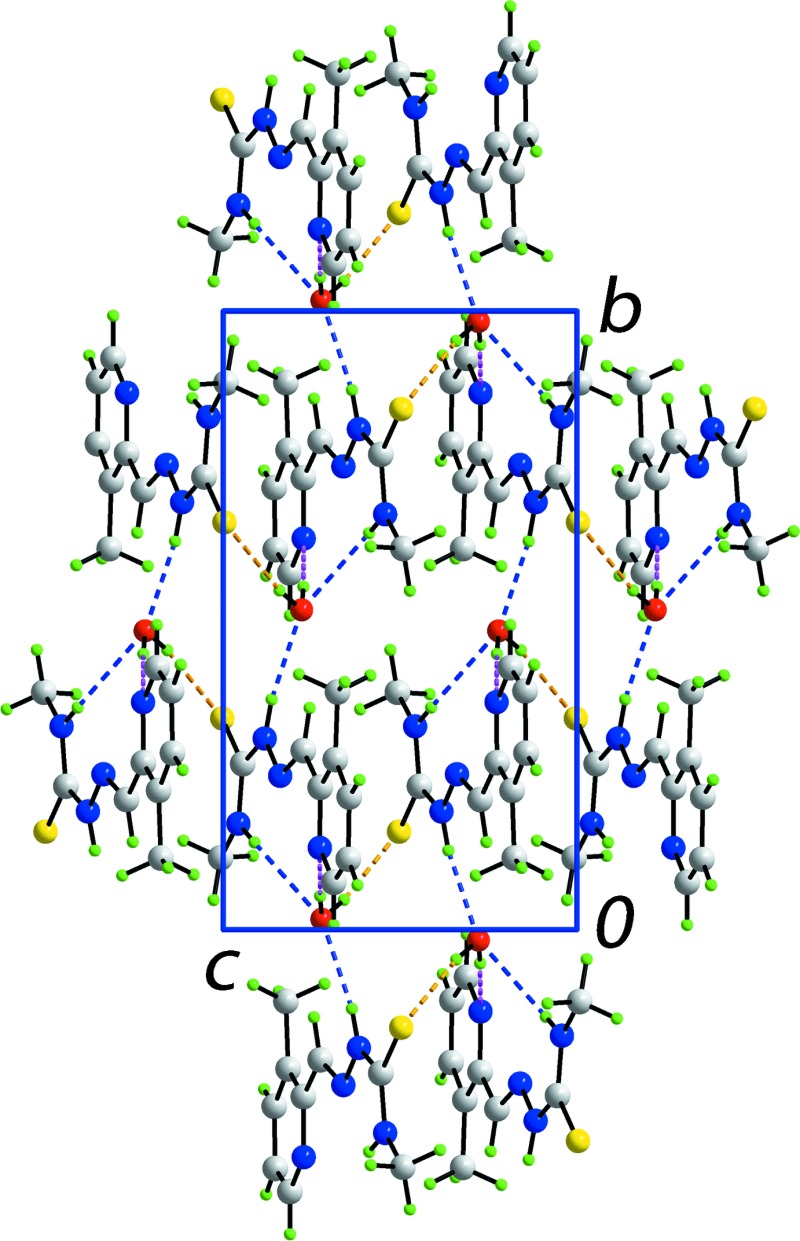
A view of the supramolecular layer in parallel to (1 0 0) sustained by N—H···O (blue dashed lines), O—H···N (pink) and O—H···S (orange) hydrogen bonding.

**Fig. 3.**
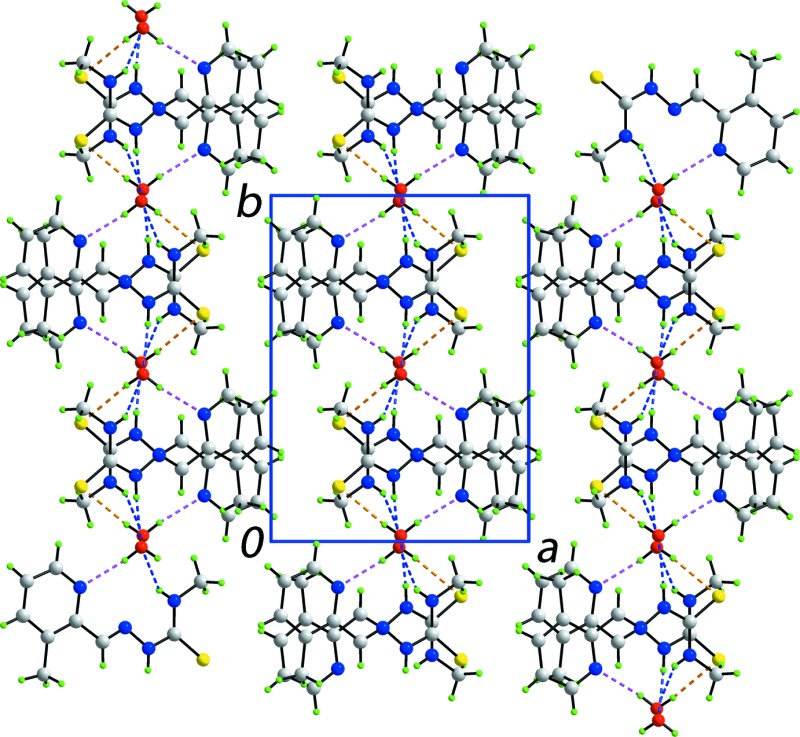
A view of the unit-cell contents in projection down the c axis. The N—H···O (blue), O—H···N (pink) and O—H···S (orange) hydrogen bonds are shown as dashed lines.

. DOI: 10.1107/S2056989015005034/hb7381fig2.tif
A view of the supra­molecular layer in parallel to (1 0 0) sustained by N—H⋯O (blue dashed lines), O—H⋯N (pink) and O—H⋯S (orange) hydrogen bonding.

c . DOI: 10.1107/S2056989015005034/hb7381fig3.tif
A view of the unit-cell contents in projection down the *c* axis. The N—H⋯O (blue), O—H⋯N (pink) and O—H⋯S (orange) hydrogen bonds are shown as dashed lines.

CCDC reference: 1053448

Additional supporting information:  crystallographic information; 3D view; checkCIF report

## Figures and Tables

**Table 1:** Hydrogen-bond geometry (, )

*D*H*A*	*D*H	H*A*	*D* *A*	*D*H*A*
N4H4NN2	0.88(2)	2.19(2)	2.6116(16)	109(1)
N1H1NO1*W* ^i^	0.88(1)	2.12(1)	2.9940(15)	170(2)
N4H4NO1*W*	0.88(2)	2.50(2)	3.3100(15)	154(1)
O1*W*H1*W*N3	0.84(2)	2.11(2)	2.9371(16)	172(2)
O1*W*H2*W*S1^ii^	0.85(2)	2.50(2)	3.3412(11)	173(2)

Symmetry codes: (i) 
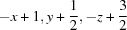
; (ii) 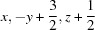.

## supplementary crystallographic information

### S2. Experimental

The Schiff base ligand was prepared according to Ali *et al.* (1997). 4-Methyl-3-thiosemicarbazide (0.01 mol) was dissolved in hot 95% ethanol (50 ml), and an equimolar amount of 3-methylpyridine-2-carbaldehyde in the same solvent (20 ml) was added. The mixture was heated with occasional stirring until the volume reduced to 20 ml. It was allowed to stand overnight and a yellow precipitate formed, which was filtered off and washed with cold ethanol. Crystals suitable for X-ray diffraction analysis were obtained by recrystallization from ethanol. Yields were high, *ca*. 92%.

### S3. Refinement

Carbon-bound H-atoms were placed in calculated positions (C—H = 0.95 to 0.98 Å) and were included in the refinement in the riding model approximation with *U*_iso_(H) = 1.2–1.5*U*_eq_(C). The O—H atoms were refined with O—H = 0.84±0.01 Å, and with *U*_iso_(H) = 1.5*U*_eq_(O). The N—H H atoms were treated similarly with N—H = 0.88±0.01 Å and *U*_iso_(H) = 1.2*U*_eq_(N).

### Crystal data

**Table:**

C_9_H_12_N_4_S·H_2_O	*F*(000) = 480
*M**_r_* = 226.30	*D*_x_ = 1.342 Mg m^−^^3^
Monoclinic, *P*2_1_/*c*	Cu *K*α radiation, λ = 1.5418 Å
*a* = 10.4493 (3) Å	Cell parameters from 7855 reflections
*b* = 13.6989 (3) Å	θ = 3.2–71.2°
*c* = 8.0235 (3) Å	µ = 2.42 mm^−^^1^
β = 102.816 (3)°	*T* = 100 K
*V* = 1119.90 (6) Å^3^	Prism, pale-brown
*Z* = 4	0.30 × 0.20 × 0.10 mm

### Data collection

**Table:**

Oxford Diffraction Xcaliber Eos Gemini diffractometer	2160 independent reflections
Radiation source: fine-focus sealed tube	2044 reflections with *I* > 2σ(*I*)
Graphite monochromator	*R*_int_ = 0.022
Detector resolution: 16.1952 pixels mm^-1^	θ_max_ = 71.4°, θ_min_ = 4.3°
ω scans	*h* = −12→12
Absorption correction: multi-scan (*CrysAlis PRO*; Agilent, 2011)	*k* = −16→16
*T*_min_ = 0.860, *T*_max_ = 1.000	*l* = −9→9
14591 measured reflections	

### Refinement

**Table:**

Refinement on *F*^2^	5 restraints
Least-squares matrix: full	Hydrogen site location: mixed
*R*[*F*^2^ > 2σ(*F*^2^)] = 0.034	H atoms treated by a mixture of independent and constrained refinement
*wR*(*F*^2^) = 0.097	*w* = 1/[σ^2^(*F*_o_^2^) + (0.0657*P*)^2^ + 0.3425*P*] where *P* = (*F*_o_^2^ + 2*F*_c_^2^)/3
*S* = 1.05	(Δ/σ)_max_ = 0.001
2160 reflections	Δρ_max_ = 0.31 e Å^−^^3^
150 parameters	Δρ_min_ = −0.25 e Å^−^^3^

### Special details

**Table:**

Geometry. All e.s.d.'s (except the e.s.d. in the dihedral angle between two l.s. planes) are estimated using the full covariance matrix. The cell e.s.d.'s are taken into account individually in the estimation of e.s.d.'s in distances, angles and torsion angles; correlations between e.s.d.'s in cell parameters are only used when they are defined by crystal symmetry. An approximate (isotropic) treatment of cell e.s.d.'s is used for estimating e.s.d.'s involving l.s. planes.

### Fractional atomic coordinates and isotropic or equivalent isotropic displacement parameters (Å^2^)

**Table:**

	*x*	*y*	*z*	*U*_iso_*/*U*_eq_	
S1	0.73687 (3)	0.84153 (2)	0.49693 (4)	0.01984 (14)	
N1	0.52525 (11)	0.80894 (8)	0.61677 (14)	0.0178 (3)	
H1N	0.5225 (16)	0.8721 (7)	0.636 (2)	0.021*	
N2	0.43392 (11)	0.74878 (8)	0.66067 (14)	0.0178 (3)	
N3	0.25907 (11)	0.63228 (8)	0.77276 (14)	0.0199 (3)	
N4	0.62430 (11)	0.67137 (8)	0.54238 (14)	0.0187 (3)	
H4N	0.5662 (14)	0.6409 (12)	0.588 (2)	0.022*	
C1	0.62415 (13)	0.76805 (9)	0.55465 (16)	0.0168 (3)	
C2	0.34637 (13)	0.78964 (9)	0.72608 (16)	0.0177 (3)	
H2	0.3482	0.8582	0.7437	0.021*	
C3	0.24297 (13)	0.73011 (10)	0.77382 (16)	0.0168 (3)	
C4	0.16527 (14)	0.57657 (10)	0.81199 (18)	0.0228 (3)	
H4	0.1760	0.5077	0.8116	0.027*	
C5	0.05263 (14)	0.61413 (10)	0.85338 (18)	0.0224 (3)	
H5	−0.0125	0.5719	0.8792	0.027*	
C6	0.03765 (13)	0.71450 (10)	0.85609 (17)	0.0211 (3)	
H6	−0.0381	0.7420	0.8848	0.025*	
C7	0.13356 (13)	0.77500 (9)	0.81675 (16)	0.0177 (3)	
C7'	0.11857 (15)	0.88417 (10)	0.8229 (2)	0.0270 (3)	
H7'1	0.0318	0.9001	0.8432	0.040*	
H7'2	0.1274	0.9125	0.7139	0.040*	
H7'3	0.1867	0.9109	0.9158	0.040*	
C8	0.72818 (13)	0.61612 (11)	0.49226 (17)	0.0225 (3)	
H8A	0.7460	0.6438	0.3871	0.034*	
H8B	0.7008	0.5479	0.4724	0.034*	
H8C	0.8079	0.6194	0.5834	0.034*	
O1W	0.49697 (10)	0.51694 (7)	0.77859 (13)	0.0232 (2)	
H1W	0.4330 (14)	0.5544 (12)	0.774 (2)	0.035*	
H2W	0.5595 (14)	0.5483 (13)	0.841 (2)	0.035*	

### Atomic displacement parameters (Å^2^)

**Table:**

	*U*^11^	*U*^22^	*U*^33^	*U*^12^	*U*^13^	*U*^23^
S1	0.0178 (2)	0.0182 (2)	0.0254 (2)	−0.00111 (11)	0.00872 (14)	−0.00060 (11)
N1	0.0179 (6)	0.0137 (5)	0.0231 (6)	−0.0011 (4)	0.0075 (5)	−0.0007 (4)
N2	0.0173 (6)	0.0177 (5)	0.0179 (6)	−0.0021 (4)	0.0030 (4)	0.0011 (4)
N3	0.0194 (6)	0.0163 (5)	0.0238 (6)	0.0013 (4)	0.0045 (5)	0.0013 (4)
N4	0.0197 (6)	0.0163 (6)	0.0210 (6)	0.0006 (4)	0.0060 (5)	0.0001 (4)
C1	0.0163 (6)	0.0182 (6)	0.0152 (6)	0.0009 (5)	0.0017 (5)	0.0002 (5)
C2	0.0181 (6)	0.0146 (6)	0.0197 (6)	−0.0005 (5)	0.0029 (5)	0.0006 (5)
C3	0.0164 (6)	0.0175 (6)	0.0154 (6)	0.0000 (5)	0.0015 (5)	0.0005 (5)
C4	0.0244 (7)	0.0159 (6)	0.0282 (7)	−0.0005 (5)	0.0061 (6)	0.0023 (5)
C5	0.0208 (7)	0.0205 (7)	0.0266 (7)	−0.0044 (5)	0.0066 (5)	0.0025 (5)
C6	0.0174 (6)	0.0238 (7)	0.0226 (7)	0.0010 (5)	0.0055 (5)	−0.0003 (5)
C7	0.0184 (6)	0.0168 (7)	0.0172 (6)	0.0002 (5)	0.0024 (5)	0.0002 (5)
C7'	0.0292 (8)	0.0172 (7)	0.0385 (8)	0.0030 (6)	0.0161 (6)	−0.0008 (6)
C8	0.0217 (7)	0.0187 (7)	0.0261 (7)	0.0051 (5)	0.0036 (6)	−0.0024 (5)
O1W	0.0207 (5)	0.0180 (5)	0.0309 (5)	0.0003 (4)	0.0055 (4)	−0.0032 (4)

### Geometric parameters (Å, º)

**Table:**

S1—C1	1.6903 (13)	C4—H4	0.9500
N1—C1	1.3633 (17)	C5—C6	1.385 (2)
N1—N2	1.3649 (15)	C5—H5	0.9500
N1—H1N	0.881 (9)	C6—C7	1.3896 (19)
N2—C2	1.2801 (18)	C6—H6	0.9500
N3—C4	1.3338 (18)	C7—C7'	1.5057 (18)
N3—C3	1.3510 (17)	C7'—H7'1	0.9800
N4—C1	1.3281 (17)	C7'—H7'2	0.9800
N4—C8	1.4513 (17)	C7'—H7'3	0.9800
N4—H4N	0.879 (9)	C8—H8A	0.9800
C2—C3	1.4706 (18)	C8—H8B	0.9800
C2—H2	0.9500	C8—H8C	0.9800
C3—C7	1.4066 (18)	O1W—H1W	0.836 (9)
C4—C5	1.391 (2)	O1W—H2W	0.848 (9)
			
C1—N1—N2	118.45 (11)	C6—C5—H5	120.8
C1—N1—H1N	121.7 (11)	C4—C5—H5	120.8
N2—N1—H1N	119.6 (11)	C5—C6—C7	119.93 (13)
C2—N2—N1	116.49 (11)	C5—C6—H6	120.0
C4—N3—C3	117.84 (12)	C7—C6—H6	120.0
C1—N4—C8	123.63 (12)	C6—C7—C3	117.47 (12)
C1—N4—H4N	115.5 (12)	C6—C7—C7'	119.96 (12)
C8—N4—H4N	119.7 (11)	C3—C7—C7'	122.58 (12)
N4—C1—N1	116.76 (12)	C7—C7'—H7'1	109.5
N4—C1—S1	124.14 (10)	C7—C7'—H7'2	109.5
N1—C1—S1	119.10 (10)	H7'1—C7'—H7'2	109.5
N2—C2—C3	119.83 (12)	C7—C7'—H7'3	109.5
N2—C2—H2	120.1	H7'1—C7'—H7'3	109.5
C3—C2—H2	120.1	H7'2—C7'—H7'3	109.5
N3—C3—C7	122.98 (12)	N4—C8—H8A	109.5
N3—C3—C2	116.66 (12)	N4—C8—H8B	109.5
C7—C3—C2	120.36 (12)	H8A—C8—H8B	109.5
N3—C4—C5	123.36 (13)	N4—C8—H8C	109.5
N3—C4—H4	118.3	H8A—C8—H8C	109.5
C5—C4—H4	118.3	H8B—C8—H8C	109.5
C6—C5—C4	118.41 (12)	H1W—O1W—H2W	102.7 (18)
			
C1—N1—N2—C2	176.76 (11)	C3—N3—C4—C5	0.1 (2)
C8—N4—C1—N1	−174.76 (11)	N3—C4—C5—C6	−0.8 (2)
C8—N4—C1—S1	5.79 (18)	C4—C5—C6—C7	0.5 (2)
N2—N1—C1—N4	−0.36 (17)	C5—C6—C7—C3	0.53 (19)
N2—N1—C1—S1	179.12 (9)	C5—C6—C7—C7'	−178.83 (12)
N1—N2—C2—C3	178.85 (11)	N3—C3—C7—C6	−1.32 (19)
C4—N3—C3—C7	1.02 (19)	C2—C3—C7—C6	178.27 (12)
C4—N3—C3—C2	−178.58 (11)	N3—C3—C7—C7'	178.02 (12)
N2—C2—C3—N3	11.03 (18)	C2—C3—C7—C7'	−2.39 (19)
N2—C2—C3—C7	−168.58 (12)		

### Hydrogen-bond geometry (Å, º)

**Table:**

*D*—H···*A*	*D*—H	H···*A*	*D*···*A*	*D*—H···*A*
N4—H4*N*···N2	0.88 (2)	2.19 (2)	2.6116 (16)	109 (1)
N1—H1*N*···O1*W*^i^	0.88 (1)	2.12 (1)	2.9940 (15)	170 (2)
N4—H4*N*···O1*W*	0.88 (2)	2.50 (2)	3.3100 (15)	154 (1)
O1*W*—H1*W*···N3	0.84 (2)	2.11 (2)	2.9371 (16)	172 (2)
O1*W*—H2*W*···S1^ii^	0.85 (2)	2.50 (2)	3.3412 (11)	173 (2)

Symmetry codes: (i) −*x*+1, *y*+1/2, −*z*+3/2; (ii) *x*, −*y*+3/2, *z*+1/2.
