# Supplementary figures and images for: Crystal structure of 3-methyl­pyridine-2-carbaldehyde 4-methyl­thio­semi­carba­zone monohydrate
Source: Acta Crystallogr E Crystallogr Commun. 2015 Mar 25;71(Pt 4):o253–4. doi: 10.1107/S2056989015005034 (PMC4438841; doi:10.1107/S2056989015005034)

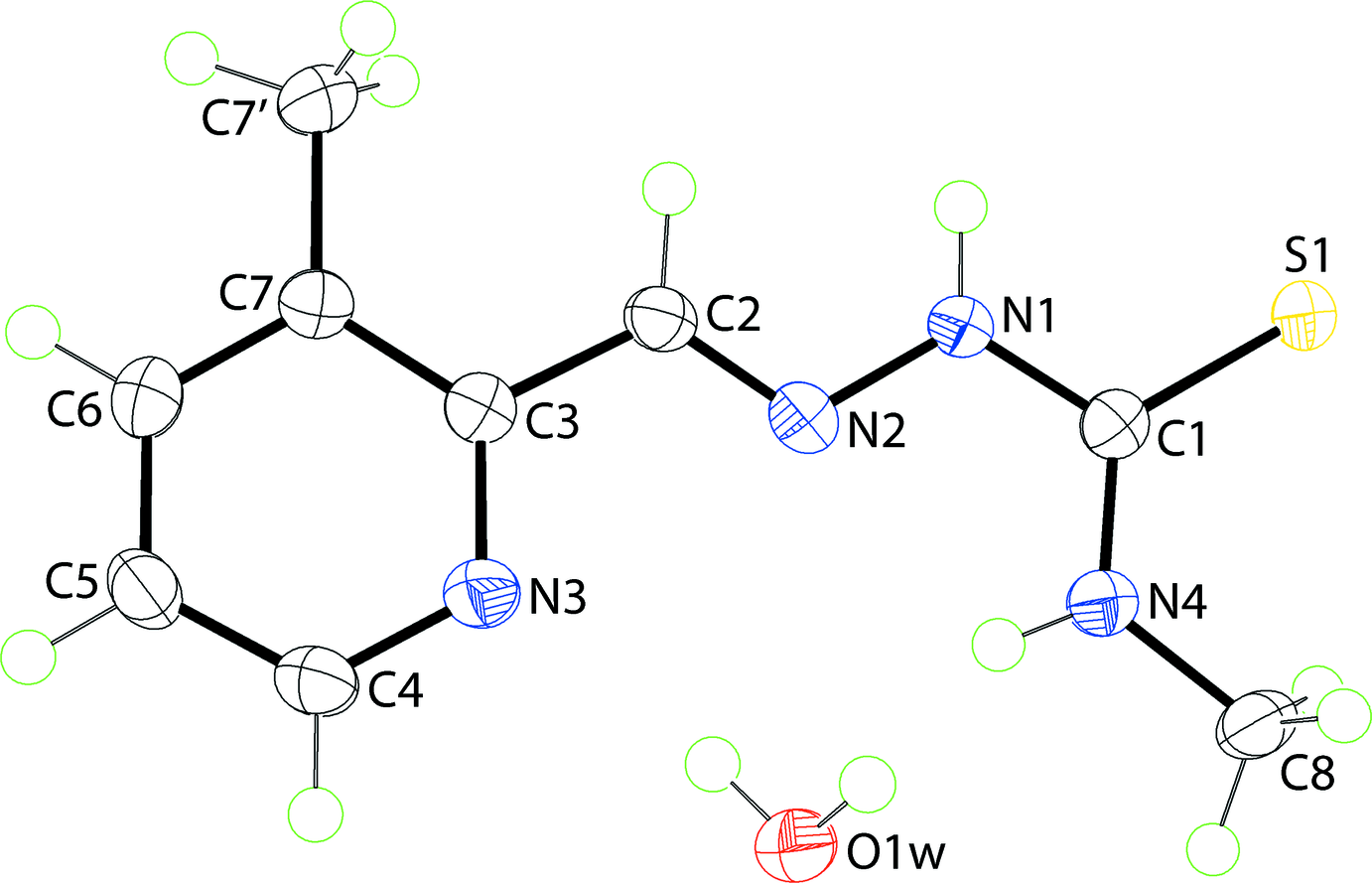

Supplement: Supplementary file 4 [file e-71-0o253-fig1.tif]

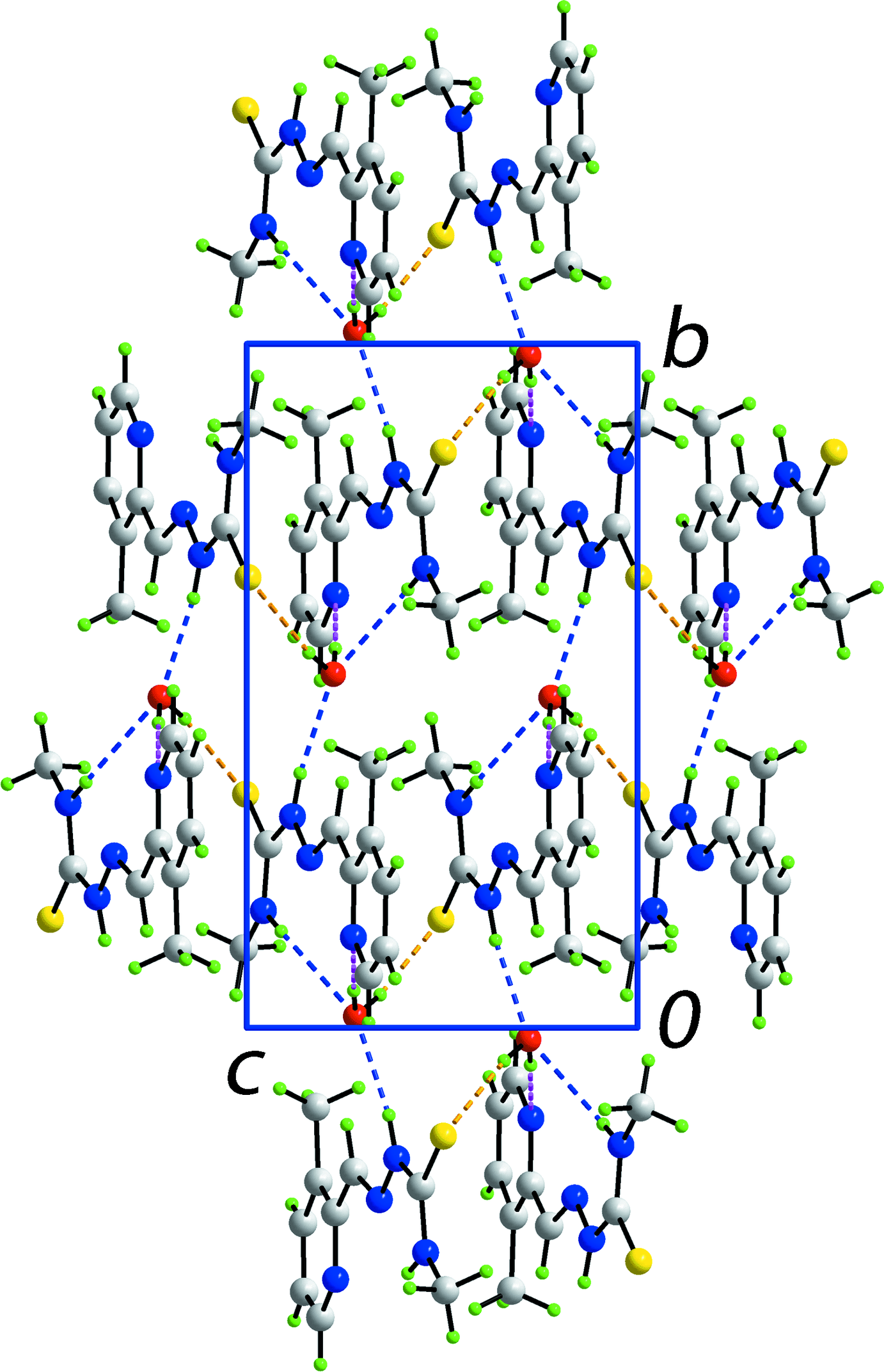

Supplement: Supplementary file 5 [file e-71-0o253-fig2.tif]

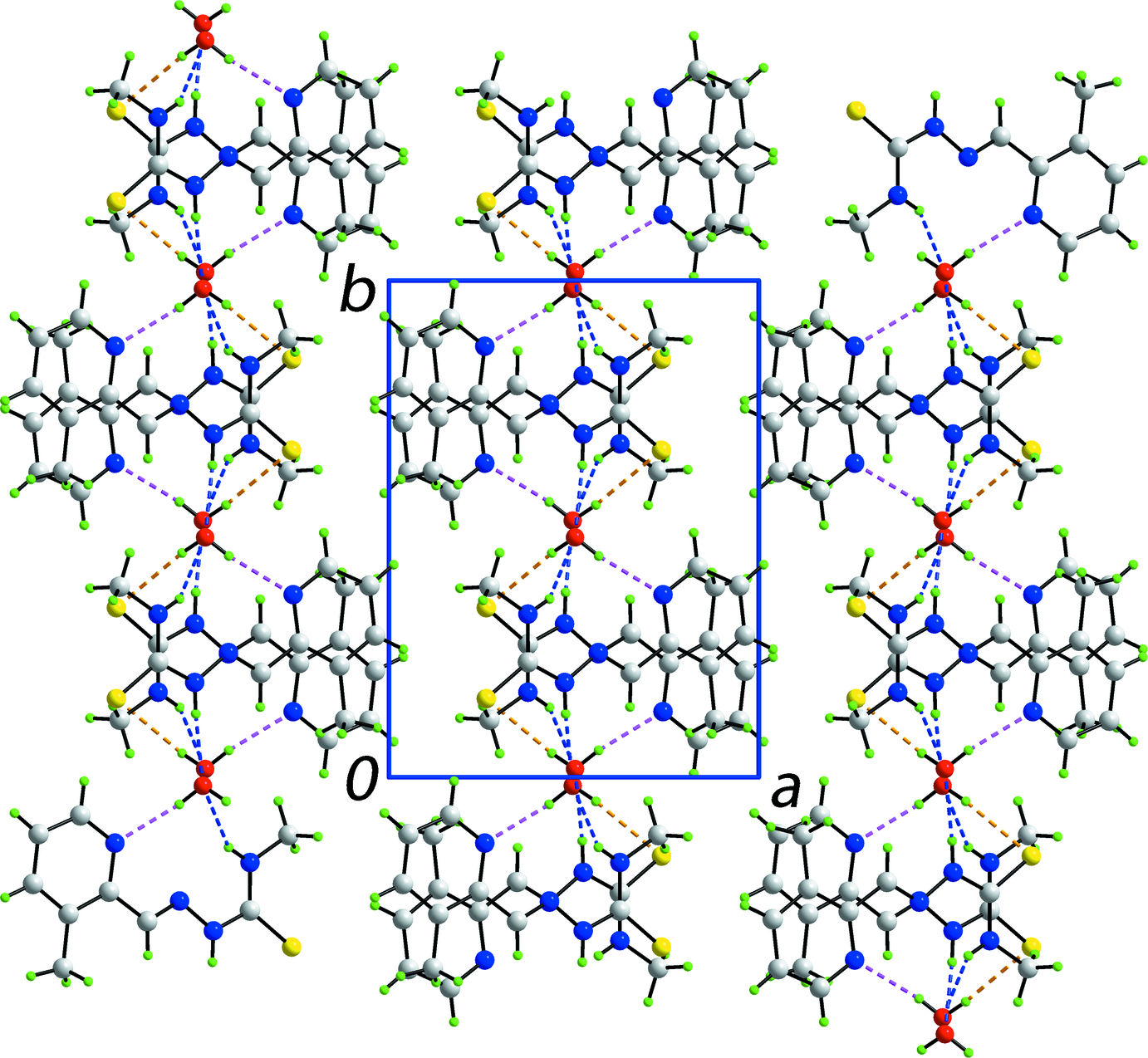

Supplement: Supplementary file 6 [file e-71-0o253-fig3.tif]
